# Structure Unveils Relationships between RNA Virus Polymerases

**DOI:** 10.3390/v13020313

**Published:** 2021-02-17

**Authors:** Heli A. M. Mönttinen, Janne J. Ravantti, Minna M. Poranen

**Affiliations:** Molecular and Integrative Biosciences Research Programme, Faculty of Biological and Environmental Sciences, University of Helsinki, Viikki Biocenter 1, P.O. Box 56 (Viikinkaari 9), 00014 Helsinki, Finland; heli.monttinen@helsinki.fi

**Keywords:** protein evolution, RNA virus, RNA-dependent RNA polymerase, protein structure, structural alignment, structure comparison, structural conservation, RNA virus taxonomy

## Abstract

RNA viruses are the fastest evolving known biological entities. Consequently, the sequence similarity between homologous viral proteins disappears quickly, limiting the usability of traditional sequence-based phylogenetic methods in the reconstruction of relationships and evolutionary history among RNA viruses. Protein structures, however, typically evolve more slowly than sequences, and structural similarity can still be evident, when no sequence similarity can be detected. Here, we used an automated structural comparison method, homologous structure finder, for comprehensive comparisons of viral RNA-dependent RNA polymerases (RdRps). We identified a common structural core of 231 residues for all the structurally characterized viral RdRps, covering segmented and non-segmented negative-sense, positive-sense, and double-stranded RNA viruses infecting both prokaryotic and eukaryotic hosts. The grouping and branching of the viral RdRps in the structure-based phylogenetic tree follow their functional differentiation. The RdRps using protein primer, RNA primer, or self-priming mechanisms have evolved independently of each other, and the RdRps cluster into two large branches based on the used transcription mechanism. The structure-based distance tree presented here follows the recently established RdRp-based RNA virus classification at genus, subfamily, family, order, class and subphylum ranks. However, the topology of our phylogenetic tree suggests an alternative phylum level organization.

## 1. Introduction

RNA polymerases are key enzymes in RNA virus replication and transcription. All currently known viral RNA-dependent RNA polymerases (RdRps) share a right-hand-shaped appearance with palm, fingers and thumb subdomains [1,2,3,4], suggesting a shared evolutionary origin. The genomes of RNA viruses, including the genes encoding the RdRp subunit, evolve fast due to the relatively high error frequency of the polymerization reaction, fast replication speed and short generation times [5].

Early sequence comparisons revealed only three strictly and two nearly conserved amino acids embedded in four short sequence motifs (A to D) to be common for viral RdRps, regardless of the nature of the viral RNA genome [6]. Additional motifs E and F were identified in some RdRps by combining sequence- and structure-based approaches [6,7,8], and finally, the weakly structured motif G was recognized using sequence-based analyses [9]. Later, these motifs were extended to homomorphs, which constitute structurally similar segments that connect template and nucleotide tunnels to the RdRp surface [10]. Initially, motifs and homomorphs were identified from RdRps of positive-strand RNA [(+)RNA] and double-stranded RNA (dsRNA) viruses, but later on, they have also been recognized in the RdRps of negative-strand RNA [(−)RNA] viruses [11]. Beyond these seven conserved motifs, there is no detectable amino acid sequence conservation among distantly related viral RdRps. This makes sequence-based comparisons of these proteins demanding. Nevertheless, stepwise sequence alignment of separate sequence clusters and subsequent analysis of inter-cluster similarities with profile-profile methods was used recently for the comparison of wide range of viral RdRp sequences [12]. Based on these data, the International Committee on Taxonomy of Viruses (ICTV) has introduced megataxonomy for RNA viruses of the realm *Riboviria* [13,14,15]. However, phylogenies based on strictly linear amino acid sequence order are not easy to infer for proteins containing internal permutations [12], such as the palm domain sequence of birnavirus and permutotetravirus RdRps, without sequence manipulation [16]. Furthermore, the lack of primary sequence similarity among distantly related RNA viruses compromises sequence alignments, making the construction of reliable long distance RdRp phylogenies demanding [17]. Therefore, additional approaches for the comparison of RdRps are needed to complement the sequence-based phylogenetic analyses and to support the implementation of higher order taxonomy for RNA viruses.

Evolutionarily related proteins typically retain a common structure and similar function, even when their sequences have diverged beyond the limits of current sequence similarity detection [18,19,20,21]. Consequently, accumulating information on protein structures can be used to reconstruct deep evolutionary relations for functionally and structurally related (viral) proteins [22,23]. Previous structure-based comparisons have revealed substantial structural conservation among the RdRps of (+)RNA and dsRNA viruses [10,24,25]. High-resolution RdRp structures from (−)RNA viruses have become available since these studies, both from viruses having segmented (families *Orhomyxoviridae*, *Peribunyaviridae*, *Arenaviridae* and *Phenuiviridae*) and non-segmented (−)RNA genome (families *Rhabdoviridae* and *Pnemoviridae*) [2,26,27,28,29,30]. All these new structures share similarities with the RdRps of (+)RNA and dsRNA viruses suggesting a common origin. In addition, the first high-resolution RdRp structure for a member of the family *Coronaviridae*, SARS-CoV-2, was determined recently [31,32] and now available for structure-based phylogenetic analyses.

Here, we studied the structural conservation among 42 RdRps, representing 16 viral families including representatives from (−)RNA, (+)RNA, as well as dsRNA viruses. The dataset includes RdRps applying different priming and transcription mechanisms. Moreover, the RdRps of permutotetravirus and birnaviruses contain a unique sequence permutation in the palm subdomain, not present in the other structurally characterized RdRps. For the structural comparison and deduction of the structure-based distance tree, we used homologous structure finder (HSF) [33], which allows comprehensive comparisons of proteins, not only within a protein family (such as RNA-dependent RNA polymerase; a SCOP identifier 4002808; [34]), but also between protein families and superfamilies, significantly extending the depth of sequence-based phylogenies [20].

We show that the currently available viral RdRps structures share a common structural core of 231 residues. In our structure-based distance tree, the polymerases are robustly clustered according to their priming mechanism, the type of primer, and the transcription mechanisms. The picornavirus and calicivirus RdRps, which use VPg-protein primer, cluster separately from the birnavirus RdRps, which use self-priming (i.e., the RdRp functions as a protein primer), and from the bunya- and orthomyxoviruses, which can apply RNA primers. Thus, the ability to use a primer probably has developed independently in each of these RdRp groups. Our results also indicate that the structurally characterized RdRps harboring sequence permutations share a common origin. Furthermore, based on our analysis, we can put forward a hypothesis that RdRps of (−)RNA viruses have evolved from an ancestral dsRNA virus polymerase, which utilized conservative transcription mechanisms, while (+)RNA virus RdRps descend from an ancestral dsRNA virus RdRps, employing semi-conservative transcription mechanisms. Overall, our results largely support the recently established RdRp-based RNA virus taxonomy and bring new insights into the functional specialization of RdRp during evolution.

## 2. Materials and Methods

The viral RdRp structures used in this study (Supplementary Table S1) were selected from the Protein Data Bank (PDB) (www.pdb.org; structures before 15th April 2020). Structures with the lowest number of disordered residues and the highest resolution were preferred. From the vesicular stomatitis Indian virus structure (PDBid: 5A22 chain A), only the polymerase domain (residues 35–865) [26] was selected for the structural alignment, as the large overall structure disturbed the alignment. Corresponding RdRp amino acid sequences for sequence alignment were obtained from PDB. 

The RdRp structures were aligned and the equivalent residues were identified using HSF [33]. We applied previously described parameters optimized for the right-hand-shaped polymerases [25], except parameter defining structural alignment was further optimized to obtain larger core and to improve the overall score for the alignment (Supplementary Table S2). For the identification of the equivalent residues, HSF performs a progressive pairwise comparison of structures. The common cores identified for pairs are further aggregated into groups (subcores) which are pair-wisely eventually merged into a final core for the entire dataset (comprises the structurally equivalent residues present in all the structures of the dataset). This results in the identification of equivalent residues for the entire dataset of structures, but also for different subsets of structures (subcores), which share higher similarity. Positionally and chemically conserved amino acids were identified using a locally written Python script [25], which searches for identical amino acids in a window of ±1 residues around the structurally equivalent amino acids. Visual Molecular Dynamics 1.9.3 [35] was used for the visualization of the common structural core. 

The structure-based distance tree was deduced based on the identified equivalent residues shared by all the structures in the dataset, i.e., the common structural core. Pairwise scores were first calculated using the core residues and parameters defined in the Supplementary Table S2. As there are currently no standard procedures for converting structural alignments into distances reflecting evolution, we converted the obtained scores using HSF [33] to distances. The distance D(A,B) between structures A and B is obtained from the pairwise score S(A,B) as D(A,B) = −(S(A,B) − min[S(A,A), S(B,B)]). This all-against-all distance matrix was converted to a tree by using the Fitch-Margoliash algorithm [36] and the resulting tree was visualized with Dendroscope 3 [37] and iTol [38]. 

Jackknifing was performed as described previously [20,21] by removing one RdRp structure at a time from each viral family, by calculating the common structural core for the partial dataset, and by constructing the structure-based distance trees from the identified new common structural core. The omitted structure for the jackknife test was selected by using a self-written python script that randomly picks one identifier from the set of all the PDB identifiers used in the study. Jackknifing trees were compared to the original tree using the ete-compare tool of ete3 package [39]. In addition, a majority rule consensus tree was made from the jackknifing trees using Dendroscope 3.

Amino acid sequence alignment was done using L-INS-i algorithm of Mafft v. 7.453 software [40].

## 3. Results and Discussion

### 3.1. Identification of a Common Structural Core for Viral RdRps

We selected 42 high-resolution viral RdRp structures from the Protein Data Bank, one from each viral species (Supplementary Table S1). These structures represent polymerases of (+)RNA viruses from the viral families *Caliciviridae*, *Coronaviridae*, *Flaviviridae*, *Leviviridae* and *Permutotetraviridae*, (−)RNA viruses of the families *Arenaviridae*, *Orthomyxoviridae*, *Peribunyaviridae*, *Pneumoviridae*, *Phenuiviridae* and *Rhabdoviridae*, and dsRNA viruses of the families *Birnaviridae*, *Cystoviridae*, *Picobirnaviridae* and *Reoviridae* (Table 1). Our dataset comprises altogether 15 new RdRp structures (Supplementary Table S1) not included in previous structure-based phylogenetic analyses of viral RdRps [6,11,24,25,41]. Most of these structures (13 RdRps) represent new viral genera, and one third of the structures were for new viral families for which there were no RdRp structures available during previous studies. The selected proteins were structurally aligned using HSF [33], which progressively compares protein structures and merges the most similar structures by identifying the equivalent residues until all the structures are part of a hierarchical clustering and the common structural core is identified for the entire dataset.

**Figure 1:**
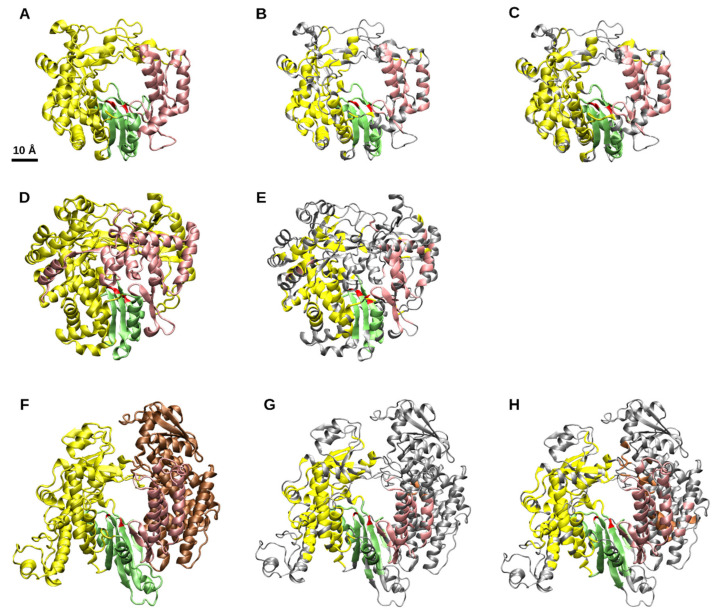
Polymerase subdomains and the common structural core of viral RNA-dependent RNA polymerases (RdRps) depicted on a (+)RNA virus (poliovirus, i.e., enterovirus **C**; **A**–**C**), a dsRNA virus (phage phi6; **D**,**E**) and a (−)RNA virus (vesicular stomatitis Indian virus; **F**-**H**) RdRps [Protein Data Bank identification codes: 3OL6, chain A; 1UVJ, chain A; 5A22 chain A (residues 35-865), respectively]. (**A**,**D**,**F**) The polymerase subdomains are indicated as yellow, fingers; green, palm; pink, thumb. Brown color is used for the N-terminal region of the vesicular stomatitis virus [26]. Red residues in the palm subdomain mark the conserved aspartic acid residues. (**B**,**E**,**G**) The 231 equivalent residues of the common structural core depicted using the same color code. Residues outside the core are in gray. (**C**,**H**) Common structural subcores of 323 and 271 residues for the two main RdRp clusters, Cluster I [panel H; comprises reovirus and (−)RNA virus RdRps] and Cluster II (panel C; comprises rest of the RdRps), respectively.

The common structural core identified for the viral RdRps is composed of 231 equivalent residues and is visualized on poliovirus [(+)RNA virus], phage phi6 (dsRNA virus) and vesicular stomatitis Indian virus [(+)RNA virus] RdRp structures in Figure 1 (Figure 1B,E,G respectively). Amino acid sequence alignment by Mafft L-INS-i across all the corresponding complete RdRp sequences failed to align the catalytic sites (Supplementary Data). This highlights the robustness of the evolutionary signal in distantly related protein structures which contain sequence permutations and share little or no sequence similarity. The average root-mean-square distance (rmsd) calculated across all of the 42 structures for the structurally aligned 231 equivalent residues was 4.51 Å. Here, the average rmsd should not be interpreted directly as similarity in direct structural comparison, as all the equivalent residues have been aligned separately to the common core per structure. Similarly to sequence-based phylogenies, a certain amount of structural diversity is required in each equivalent position, so that further structure-based comparison is meaningful.

None of the amino acid residues assigned to be equivalent by HSF were chemically identical. However, the conserved catalytic aspartates (depicted in Figure 1) could be identified from equivalent positions if the positional constraints were slightly reduced (identical residues searched in a ±1 sequence window; [25]). Thus, the key catalytic residues are in similar positions relative to the core structure, irrespectively of RdRp origin and its specific structural and functional features.

**Figure 2:**
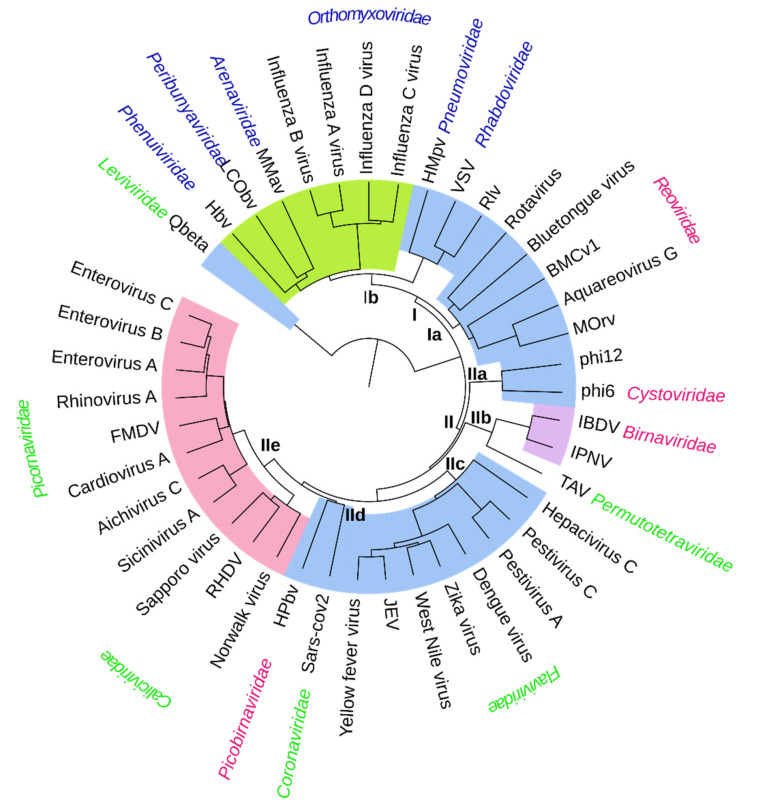
A structure-based phylogenetic tree for the polymerase subunits of RNA viruses deduced based on the structural alignment of the common core. The name of the virus (black) and the viral family are indicated at the tips of the branches. The coloring of the family name reflects the nature of the viral genome: green, positive-strand RNA; blue, negative-strand RNA; and red, double-stranded RNA. The polymerases using solely primer-independent initiation are indicated with blue background, polymerases using VPg-protein primer with pink background, polymerases applying self-priming with purple background, and polymerases using RNA-priming on (+)strand synthesis with green background. Background is white if the initiation mechanism is unclear. Moreover, in this case, there is biochemical and structural evidence that suggests a primer-independent initiation mechanism [43]. The applied abbreviations are: BMCv1: Bombyx Mori cypovirus 1, FMDV: foot-and-mouth disease virus, Hbv: Huaiyangshan banyangvirus, HPbv: human picobirnavirus, HMpv: human metapneumovirus, IBDV: infectious bursal disease virus, IPNV: infectious pancreatic necrosis virus, JEV: Japanese encephalitis virus, LCObv: La Crosse orthobunyavirus, MMav: Machupo mammarenavirus, MOrv: mammalian orthoreovirus, phi6: Pseudomonas phage phi6, phi12: Pseudomonas phage phi12, Qbeta: Escherichia phage Qbeta, RHDV: rabbit haemorrhagic disease virus, Rlv: rabies lyssavirus, SARS-CoV-2: severe acute respiratory syndrome coronavirus 2, TAV: thosean asigna virus, VSV: vesicular stomatitis Indian virus.

The identified 231-residues common structural core covers the catalytic site at the palm subdomain and large regions in the fingers subdomain as well as three α-helices in the thumb subdomain (Figure 1B,E,G). The identified equivalent residues also overlap well with the previously identified sequence motifs A to E and the corresponding homomorphs (Supplementary Figure S1; Supplementary Figure S2B,E). However, due to the gaps, e.g., in the RdRp structures of Zika virus (PDBid 5WZ3) and Machupo mammarenavirus (PDBid 6KLD), at the regions of motif F and G, respectively, structural equivalence could not be identified in the area of motif G and the core covered only few residues at the motif F boundaries (Supplementary Figures S1 and S2). The initial clustering of the RdRps by HSF resulted in two main clusters and the identification of common, structurally equivalent residues for the members of these clusters (i.e., Cluster I and II subcores, Figure 1H,C, respectively). Cluster I comprises RdRps from the members of the *Reoviridae* family and all the RdRps of (−)RNA viruses included in our dataset. Cluster II contains all the RdRps of (+)RNA viruses and the remaining dsRNA virus RdRps (see Branch I and II in Figure 2). The sizes of these cores are 323 (average rmsd 4.2 Å) and 271 (average rmsd 4.2 Å) residues for the Cluster I and II, respectively. The additional equivalent residues within these larger cores mostly fill in small gaps present in the 231-residue core (Figure 1B,E,G) identified for all the RdRp structures. Moreover, residues of motif G were partially covered by the Cluster II core and more structural equivalence could be observed in Cluster I core at the motif F area than was initially detected in the core of the complete dataset (Supplementary Figure S2). Furthermore, additional structurally conservation is detected among (−)RNA and reovirus RdRps (Cluster I) in the N-terminal region (compare Figure 1G,H).

The identified 231-residues common structural core of viral RdRps covers over 30% of the influenza A virus, over 50% of the poliovirus and 25% of SARS-CoV-2 polymerase structures. Thus, despite the minimal sequence similarity, the structural conservation across all the viral RdRp structures is substantial. This implies that the identification of target sites for wide-spectrum antivirals could be possible. The high structural conservation also supports the hypothesis that all the viral RdRps, including those from (−)RNA viruses, descend from a common ancestor.

### 3.2. Structure-Based Distance Tree and Overall Grouping of the RdRp Structures

**Figure 3:**
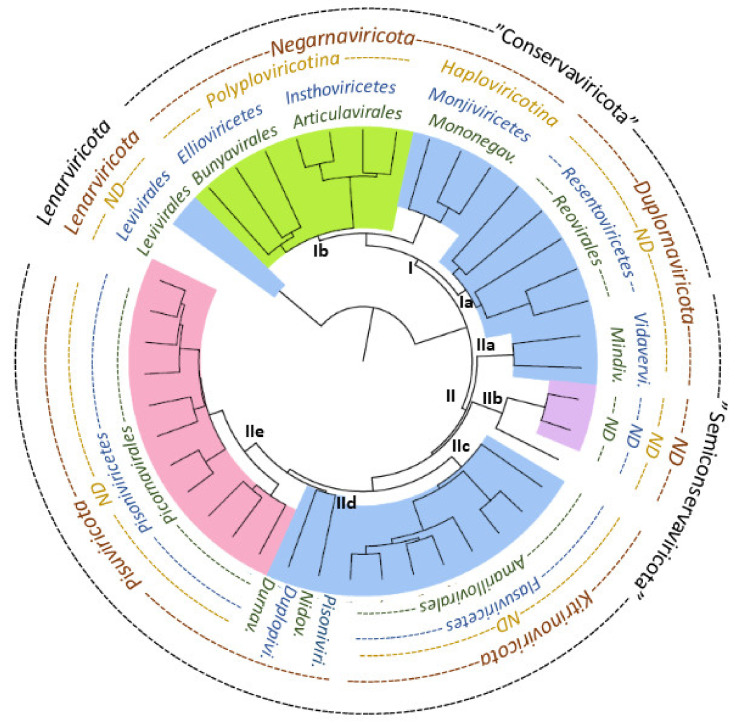
The higher order taxonomy of RNA viruses at order, class, subphylum and phylum ranks depicted on the structure-based distance tree. The different ranks are on green, blue, dark yellow and maroon fonts, respectively. The phylum level classification proposed here is on black font. For branch background colors, please see Figure 2. Abbreviations *v*. and *vi*. refer to *virales* and *viricetes*, respectively.

In order to obtain further insights into the structural relationships among viral RdRps, the identified common structural cores were compared between different polymerase structures using HSF [33] and a structure-based distance tree was reconstructed by comparing the properties of the core region in the individual RdRp structures (Figure 2). Based on earlier structure- and sequence-based phylogenetic analyses, the levivirus RdRps likely have diverged early from the common ancestor of the other RdRps [12,41], and therefore, the bacteriophage Qbeta RdRp structure was used here as an outgroup for rooting. In the resulting tree (Figure 2), the clustering of structures accurately follows the well-established genus, subfamily and family classification of the corresponding viruses (Table 1) but also the new higher order taxonomy at order, class and subphylum ranks (Figure 3; Table 1; [14]).

In our structure-based distance tree, the viral RdRps form two major branches (I and II; Figure 2). The reovirus RdRps are in the base of Branch I, while the cystovirus RdRps form the base of Branch II. These dsRNA virus RdRps, together with the levivirus RdRp, used here as an outgroup for rooting, have been also previously proposed to represent the earliest RdRp forms diverged from the common ancestor [24,41]. In addition to reovirus RdRps, Branch I contains all the (−)RNA virus RdRps included in our analyses. The (−)RNA virus RdRps form a sister branch (Subbranch Ib) for the reovirus RdRp branch (Subbranch Ia). The subbranch of (−)RNA virus RdRps is composed of two main subgroups, representing RdRps from non-segmented and segmented (−)RNA viruses. Such division could not have been clearly demonstrated in earlier structure-based phylogenetic analyses [11,44]. Branch II contains all the (+)RNA virus RdRps (except levivirus RdRp) as well as the remaining dsRNA viruses. In addition to the cystovirus RdRp subbranch (IIa), two subbranches of (+)RNA viruses can be identified in Branch II (IIc and IIe) and two mixed subbranches containing RdRps from both (+)RNA and dsRNA viruses (Subbranches IIb and IId).

Thus, while the (−)RNA virus RdRps appear to be a monophyletic group (Figure 2; [12]), the dsRNA and (+)RNA virus RdRps could be paraphyletic or polyphyletic, based on our analysis and previous studies [12]. Such observation indicates that evolution of the viral RdRps does not necessarily follow the type of the viral genome, but transition from double-stranded to single-stranded RNA genome, or vice versa, might have independently occurred in viruses associated with different RdRp lineages (i.e., different subbranches) [12,41]. While the majority of the dsRNA virus RdRps are located close to the root of the tree, including reoviruses, cystoviruses and birnaviruses, the representative RdRp structure from picobirnaviruses is located together with the coronavirus RdRp structure in a larger branch containing major subgroups of (+)RNA viruses. Similar separation of picobirnavirus RdRp from the other dsRNA virus RdRps has also been documented in sequence-based phylogenetic analyses [12], where also partitivirus RdRps were shown to cluster with picobirnavirus RdRps. The capsids of picobirna- and partitiviruses are related to the inner capsids of reoviruses and cystoviruses. The 120-subunit capsid organization shared by these (and many other) dsRNA viruses is unique and not observed in (+)RNA or in any other viruses [45,46,47,48]. This rules out an evolutionary scenario in which picobirnaviruses and partitiviruses would simply be developed from a (+)RNA virus (or vice versa) due to a change in the genome encapsidation process. Consequently, recombination or reassertion between the RdRp and capsid genes of (+)RNA and dsRNA viruses has been proposed [12,49].

In the case of dsRNA viruses (including picobirnaviruses and partitiviruses), the polymerase is an integral part of the viral capsid and it needs to interact correctly with the capsid protein during the virion assembly and the subsequent intra-capsid genome replication and transcription processes [50,51,52]. These functional constraints likely restrict the formation of functional new capsid-RdRp gene combinations in dsRNA virus genomes. In most known (+)RNA viruses, the genome replication is instead spatially and temporarily separated from the virion assembly, making new capsid-RdRp combinations more likely to be viable. Based on this, the conservation of the capsid, and the overall topology of the structure-based distance tree, we suggest that picobirnaviruses RdRp and capsid genes have developed directly from the ancestral cysto/reovirus-type of dsRNA viruses. Interestingly, structure-based clustering of the dsRNA virus 120-subunit capsid proteins [47] correlates well with the RdRp phylogenies (Figure 2; [12]) of the corresponding viruses. This suggests a tight linkage between these two proteins (co-evolution) and supports the evolutionary scenario for picobirnaviruses presented here.

### 3.3. Stability of the Structure-Based Distance Tree and the Common Structural Core

A simplified jackknifing test was carried out to evaluate the robustness of the structure-based distance tree. The overall topology of the tree (Figure 2) was maintained in all the jackknifing replicates and the deduced jackknifing consensus tree (Supplementary Figure S3A) was identical with the tree presented in Figure 2. Slight variation in the branching was observed only in two repetitions (Supplementary Figure S3B and C; Supplementary Table S3).

The jackknifing test also provides information on the stability of the core size as the equivalent residues, i.e., the common structural core is calculated separately for each of the partial dataset as part of the test ([20,21]; see Methods). No notable effect on the size of the common structural core (219−240 residues) or the rmsd (4.4−4.8 Å) (see Supplementary Table S3) was observed, even in those eight cases where the only representative structure from a viral family was withdrawn from the dataset. The obtained core size was also very close to that obtained previously (260 residues; rmsd 4.6 Å [25]) with a substantially smaller dataset comprising only 18 RdRps, including no representatives from (−)RNA viruses or families *Picobirnaviridae*, *Coronaviridae*, and *Permutotetraviridae*. Consequently, none of the individual RdRp brings substantial additional structural diversity to the dataset, implying that the structural diversity in the core structure is relatively constant across the studied viral RdRps. Notably, the overall structural architecture of the palm subdomain appears to be unaffected by the different connectivity in the birnavirus and permutotetravirus RdRps [29,43,53], allowing the alignment and comprehensive analysis of all the available RdRp structures.

### 3.4. RdRps from (+)RNA and dsRNA Viruses with Permutated Catalytic Site Sequence Group Together

Birnaviruses and permutotetraviruses represent dsRNA and (+)RNA viruses, respectively. Yet, their RdRps are structurally similar and contain a unique sequence permutation, not seen in any other known structurally characterized viral RdRps, suggesting shared origin [9,43]. The linear order of amino acids has little influence on the structure-based phylogenetic analysis presented here due to the selected parameters, which emphasize structural and physicochemical properties, like geometry, secondary structure and amino acid properties, rather than amino acid sequence order (Supplementary Table S2). Nevertheless, in our structure-based distance tree, birnavirus and permutotetravirus RdRps are both located in the IIb Subbranch (Figure 2; Supplementary Figure S3). This suggest that there are evolutionary signals in these structures, other than the sequence permutation, that make them group together. The result supports the earlier observations on evolutionary connection between birna- and permutotetravirus RdRps [9,43].

Birnaviruses are unique among dsRNA viruses as they lack the characteristic 120-subunit capsid organization of other dsRNA viruses. Instead, birnaviruses have a 780-subunit capsid organized into T = 13 icosahedral lattice as trimers of the major capsid protein [54]. Interestingly, the 120-subunit inner capsid of reoviruses and cystoviruses is enclosed into a shell that shares the same T = 13 *laevo* arrangement [48,55,56]. The mainly α-helical cystoviral T = 13 shell protein represents the simplest [56] and likely the most primitive form of the current T = 13 shell proteins of dsRNA viruses. Yet, it shares the key functionalities with the eukaryotic dsRNA virus counterparts by facilitating the delivery of the internal ribonucleoprotein complex through membrane of an endocytic like vesicle during virus entry [57]. The birnavirus and reovirus T = 13 shell proteins both have an α-helical basal domain and a structurally similar radially oriented “jelly roll” β-barrel projector decorating the base [54]. Based on the structural relatedness, two alternative evolutionary scenarios have been presented, in which birnaviruses either have lost the characteristic inner capsid of dsRNA viruses during their evolution from an ancestral reovirus, or reoviruses have obtained the outer T = 13 shell from a birnavirus [54]. Considering that cystoviruses infecting bacterial host also have the T = 13 *laevo* shell which has a similar role during virus entry, we favor the evolutionary pathway from a cysto/reovirus ancestor to birnavirus. Interestingly, the birnavirus and permutotetravirus capsid proteins also display substantial structural similarity, despite their different icosahedral arrangements in virions (T = 13 and T = 4, respectively) [54]. This links the permutotetravirus T = 4 capsid protein to the dsRNA virus T = 13 shell protein lineage. 

Authors of previous studies have suggested alternative scenarios for the development of birnavirus and permutatetravirus RdRps, favoring the development of birnavirus RdRp from an ancestral permutotetravirus RdRp [43,58]. Taking into account the topology of the structure-based distance tree (Figure 2), the likely evolutionary pathway of the capsid proteins, and assuming that transitions between different genome types are rare, we propose an alternative evolutionary pathway in which permutotetraviruses have descended from an ancestral birnavirus. In such a scenario, the division between the birnavirus and permutotetravirus RdRp branches would have occurred prior to the introduction of the unique ADN sequence in the place of the canonical GDD sequence [58] in the catalytic site of the birnavirus RdRps and the adoption of the protein-priming initiation mechanism by birnaviruses.

The sequence permutation in birnavirus and permutotetravirus RdRps has hampered the use of sequence-based comparison methods in the classification of these RdRps, and therefore there is no established higher order taxonomy for the families *Birnaviridae* and *Permutotetraviridae* of the kingdom *Orthornavirae* (Table 1). A recent sequence-based analysis, however, suggests that these RdRps are related to the picobirnavirus RdRps, while other RdRp sequences with permuted catalytic sites were shown to group close to flavivirus RdRps suggesting convergent evolution of this feature [16]. Such analysis was enabled by swapping the permuted RdRp domains in the sequences to restore the conventional domain order, allowing multiple sequence alignment. In our structure-based distance tree, permutotetravirus and birnavirus RdRps form a separate subgroup (IIb) which branches from the node giving rise to both flavivirus and picobirnavirus RdRps (Figure 2).

### 3.5. Early Separation of Semi-Conservative and Conservative Mechanism of Viral RNA Transcription

Nucleic acid polymerases may produce new single-stranded molecules from double-stranded templates, using either conservative or semi-conservative mechanisms. Reovirus RdRps use conservative transcription mechanisms [4,59,60], while the RdRps of other dsRNA viruses included in this study (i.e., cystoviruses, birnaviruses and picobirnaviruses) apply semi-conservative transcription [53,61,62]. This difference is reflected in the structures of these RdRps. Reovirus RdRp has four tunnels: (i) for the template entry, (ii) for the NTP entry, (iii) for the exit of the parental negative strand which will be hybridized on the RdRp surface with the parental positive-strand, and (iv) for the exit of the nascent positive-strand [4,59]. Those dsRNA virus RdRps which use semi-conservative transcription mechanism have smaller structures with only three tunnels: (i) for the template entry, (ii) for the NTP entry, and (iii) for the exit of dsRNA formed of the parental negative-strand and nascent positive-strand [1,53,62]. Interestingly, the (−)RNA virus RdRps, that in our structure-based phylogenetic tree are located together with the reovirus RdRps in Branch I, also have a four tunnel structure [27,29,63], while the (+)RNA RdRps located in Branch II have 3-tunneled structure similar to the dsRNA virus RdRps from the *Cystoviridae*, *Picobirnaviridae*, and *Birnaviridae* [11,31,64,65,66]. Furthermore, semi-conservative transcription has been documented for the RdRps of flaviviruses and picornaviruses [67,68]. These observations suggest that the 3- and 4-tunnel RdRps (applying semi-conservative and conservative transcription mechanisms, respectively) have separated early (Branches I and II; see also [11]) and these properties have monophyletic origin. Building on this hypothesis, we can predict that partitivirus and togavirus RdRps, which based on existing biochemical evidence, use a semi-conservative transcription mechanism [69,70], belong to Branch II.

### 3.6. Branch I: Origin of (−)RNA Virus RdRps and Development of RNA-Primed Initiation

The RdRps of (−)RNA viruses with segmented and non-segmented genomes (*Polyploviricotina* and *Haploviricotina*, respectively, in our data set; Figure 3) are clustered in the sister Subbranch Ib of reovirus RdRps Subbranch Ia (Figure 2). This association of branches is strong and maintained unchanged in all the jackknifing replicates (see Supplementary Figure S3), suggesting that the RdRps of (−)RNA viruses share a common origin with the RpRps of dsRNA viruses from the *Reoviridae* family. The observation is also in line with the earlier sequence-based phylogenetic analysis [12], which predicted that RdRps of (−)RNA viruses form a monophyletic group that roots from a branch composed of reoviral and cystoviral RdRps, and the structure-based analysis where (-)RNA RdRps were shown to form a branch close to the cluster of levi- and reovirus RdRps [41,44]. 

Within our structure-based phylogenetic tree, the rhabdovirus RdRp groups together with RdRps of reoviruses (family *Reoviridae*) and segmented (−)RNA viruses (subphylum *Polyploviricotina*) may thus represent an intermediate structure between these two groups (Figure 2). Rhabdovirus and reovirus RdRps both apply primer-independent de novo initiation mechanism for replication and transcription [26,71,72,73]. The RdRps of segmented (−)RNA viruses (e.g., influenza virus and bunyavirus) also use primer-independent de novo initiation mechanism for replication, but for transcription, they pinch a cap from host messenger-RNA (cap-snatching; [71,72,74,75,76,77]) and use this short RNA strand as a primer for transcription. Interestingly, the rhabdovirus RdRp lacks the initiation loop structure commonly found from RdRps using de novo initiation and it uses a separate protein component (a loop in the capping enzyme) to stabilize the initiation complex for primer-independent RNA synthesis [78]. The deletion of the initiation loop and its functional replacement has likely been an essential step in the development of the RNA-primed RNA synthesis. Thus, the subgrouping and branching of (−)RNA virus RdRps in the structure-based phylogenetic tree follow their functional differentiation.

### 3.7. Branch II: Development of Protein Priming

The viral RdRps dependent on a protein primer are located in two different subbrances (IIb and IIe) of Branch II (Figure 2, pink and purple backgrounds). The first subbranch (IIe) is formed by RdRps which use a short polypeptide, VPg, as a primer. These RdRps are from the members of the viral families *Picornaviridae* and *Caliciviridae* and this grouping is stable in all the jackknifing trees (see Supplementary Table S3). The other subbranch (IIb) contains birnavirus RdRps which use self-priming. These two subbranches are separated in all the jackknifing replicates, suggesting that the ability to use a protein primer has developed separately for birnaviruses and for picorna- and caliciviruses. Based on the topology of the tree, both these RdRp groups have developed from ancestral RdRps that were primer-independent (Figure 2).

The protein priming mechanism of birnavirus is unique among known RNA viruses; the RdRp itself is used as a primer and consequently the polymerase is attached to the 5′-end of the genomic positive-sense strand [79]. However, the negative-sense strand of the birnavirus genome does not contain a covalently linked protein, implying that the synthesis of negative-strand initiates de novo. Notably, also caliciviruses have the ability to initiate replication without a protein primer [80]. Moreover, all the known (−)RNA virus RdRps initiate synthesis of the negative-strand de novo. Thus, the capability to initiate RNA synthesis de novo is apparently an ancient feature shared by most, if not all current viral RdRps, despite their potential alternative priming activities.

### 3.8. Considerations on the RNA Virus Taxonomy

ICTV has recently approved the possibility to classify viruses based on sequence information only, and introduced taxonomic hierarchy from order to realm [81,82]. The megataxonomy of DNA viruses is based on the observation that distantly related DNA viruses may share a similar major capsid protein fold, allowing a classification scheme which is based on this key virion component [14,22]. In the case of RNA viruses, the polymerase gene has been selected as the hallmark gene for classification. 

The sequences of viral RdRps are highly divergent, making sequence alignment and recognition of phylogenetic signals between distantly related RdRps demanding [17,83] (Supplementary Data). However, the emerging structural information on RdRps indicates that viral RdRps share a common fold, supporting the hypothesis of their common origin. Using HSF we could structurally align determine 231 equivalent residues in the available RdRp structures which represent (+)RNA, (−)RNA and dsRNA viruses. Based on this identified common structural core (Figure 1), it was possible to deduce a structure-based distance tree (Figure 2) with high confidence (Supplementary Figure S3) and compare the obtained structure-based classification with the current sequence-based RNA virus classification (Table 1; Figure 3). The grouping of the RdRp structures accurately followed the established virus taxonomy at the genus, subfamily and family levels (Figure 2). The structure-based distance tree presented here also follows the RNA virus taxonomy at the more recently established higher taxonomic ranks, including order, class and subphylum (Figure 3), thus providing further support for the earlier sequence-based analyses used to establish the current taxonomy [12]. However, the topology of our phylogenetic tree suggests an alternative phylum level organization where the RdRps using conservative transcription mechanism (Cluster I) would constitute a phylum ”Conservaviricota” and those using semi-conservative transcription (Cluster II) another phylum “Semiconservaviricota”, in addition to the already established *Lenaviricota* phylum. As a consequence of this, the current phyla *Kitrinoviricota* and *Pisuviricota* could form subphyla “Kitrinoviricotina” and “Pisuviricotina” in the “Conservaviricota” phylum. In addition, the current phylum *Negarnaviricota* could form “Negarnaviricotina” subphylum in the “Semiconservaviricota” phylum. Such classification would eventually also split the dsRNA viruses of the phylum *Duplornaviricota* into two subphyla. 

The RdRp-based RNA virus classification allows a comprehensive comparison of all RNA viruses. However, the data presented here and earlier by Wolf et al. [12] illustrate the debatable nature of such a classification scheme. DsRNA viruses of the families *Reoviridae*, *Cystoviridae*, *Picobirnaviridae*, *Totiviridae*, *Crysoviridae*, *Partitiviridae* and *Quadriviridae* form a uniquely uniform group among RNA viruses, sharing a similar capsid organization not observed in any other known virus group and common intracapsid genome replication and transcription strategies [45,46]. Yet, in the RdRp-based classification (Figure 3; [12]), these viruses are split into several different subphyla and phyla. If RNA virus classification would use similar criteria as established for DNA viruses, all these dsRNA viruses would be placed in a single monophyletic group, which would better serve the needs of the dsRNA virus community. As the sequence similarity in capsid proteins and many other RNA virus proteins is nearly undetectable, we see structure-based phylogenies for different groups of RNA virus proteins as a beneficial method to further develop the classification criteria and assign them to different virus groups.

### 3.9. Evolutionary Implications and Future Directions

De Farias et al. [84] proposed that RdRps originate from the translation products of ancient proto-tRNA molecules. They showed that the RdRps of leviviruses, reoviruses and cystoviruses are structurally most similar to such proto-tRNA translation products, implying their ancient origin [41,84]. Building on this hypothesis and data presented here, we propose that eukaryotic (−)RNA and (+)RNA virus RdRps have developed from the precursors of current dsRNA virus RdRps (supported by [8] and [24], respectively). Furthermore, RdRps capable of using protein-, RNA- or self-priming have evolved independently from RdRps that primarily use de novo initiation mechanism. This supports the assumption that RdRp catalyzed reactions have evolved from simple to more complex ones and that primer-independent RNA polymerization and polymerases would have evolved prior to the primer-dependent systems (evolution from two to three component systems). Our data also imply that transition between the three- and four-tunneled RdRp has happened only once, and that RdRps using semi-conservative transcription descend from a cystoviral type of three-tunneled RdRps, while the four-tunneled RdRps originate from the reovirus type RdRps.

Overall, our structure-based phylogenetic tree reflects key functional properties imposing structural constraints. Interestingly, these differences can be detected by comparison of the conserved core structure which does not contain features that contribute to these different functions (e.g., priming loop). Such observation suggests that a protein structure evolves little by little over generations similarly to amino acid or nucleotide sequences. The results obtained here also correlate well with earlier sequence-based analyses on viral RdRps. This indicates that the evolutionary structural signal in the three-dimensional structure of the proteins is sufficiently robust to capture differences, even between relatively similar protein structures (e.g., RdRps from viruses belonging to the same virus family), although structure-based comparison methods are generally expected to be more powerful in the identification of longer distance relationships, e.g., between protein families or superfamilies [20], than sequence-based comparison methods. This observation also confirms that the structural and sequence signals used for comparative analyses of proteins and protein phylogenies correlate well and can complement each other [20,21,24]. Moreover, structure-based analyses are directly capable of handling proteins which connectivity has altered via sequence permutations, such as in birnavirus RdRps. The direct analysis of permuted sequences and complete proteins without a requirement for sequence editing or extensive shortening is beneficial, as it minimizes the risk of inaccuracies arising from additional steps in the analysis. However, the limitation of structure-based phylogenetic analyses is the limited amount of structural information that we currently have, and that the available data is strongly biased towards medically important viruses [20,21,24]. Moreover, gaps in regions presenting structural flexibility affect the completeness of the structural alignment. However, recent improvements in computational methods, like AlphaFold that directly predicts 3D-structural models from amino acid sequence [85], may allow for the application of structure-based phylogenies for a wider spectrum of RdRps and other viral proteins in the future.

## Figures and Tables

**Table 1:** Viral origin of RNA-dependent RNA polymerase structures used in this study and the taxonomy and genome type of the viruses ^1^.

Genome	Kingdom	Phylum; Subphylum	Class; Order	Family; Subfamily	Genus	Virus
(−)RNA	*Orthornavirae*	*Negarnaviricota; Polyploviricotina*	*Ellioviricetes; Bunyavirales*	*Arenaviridae*	*Mammarenavirus*	Machupo mammarenavirus
				*Peribunyaviridae*	*Orthobunyavirus*	La Crosse orthobunyavirus
				*Phenuiviridae*	*Bandavirus*	Huaiyangshan banyangvirus
			*Insthoviricetes; Articulavirales*	*Orthomyxoviridae*	*Alphainfluenzavirus*	influenza A virus
					*Betainfluenzavirus*	influenza B virus
					*Gammainfluenzavirus*	influenza C virus
					*Deltainfluenzavirus*	influenza D virus
		*Negarnaviricota*; *Haploviricotina*	*Monjiviricetes; Mononegavirales*	*Pneumoviridae*	*Metapneumovirus*	human metapneumovirus
				*Rhabdoviridae*	*Lyssavirus*	rabies lyssavirus
					*Vesiculovirus*	vesicular stomatitis Indian virus
dsRNA		*Duplornaviricota*	*Resentoviricetes; Reovirales*	*Reoviridae; Sedoreovirinae*	*Rotavirus*	rotavirus A
					*Orbivirus*	bluetongue virus
				*Reoviridae; Spinareovirinae*	Aquareovirus	aquareovirus G
					*Cypovirus*	Bombyx mori cypovirus 1
					*Orthoreovirus*	mammalian orthoreovirus
			*Vidaverviricetes; Mindivirales*	*Cystoviridae*	*Cystovirus*	Pseudomonas phage phi6
						Pseudomonas phage phi12
		*Not determined (ND)*	*ND*; *ND*	*Birnaviridae*	*Avibirnavirus*	infectious bursal disease virus
					*Aquabirnavirus*	infectious pancreatic necrosis virus
(+)RNA				*Permutotetraviridae*	*Alphapermutotetravirus*	thosean asigna virus
		*Kitrinoviricota*	*Flasuviricetes; Amarillovirales*	*Flaviviridae*	*Hepacivirus*	hepacivirus C
					*Pestivirus*	pestivirus A
						pestivirus C
					*Flavivirus*	Dengue virus
						Zika virus
						West Nile virus
						Japanese encephalitis virus
						Yellow fever virus
ds		*Pisuviricota*	*Duplopiviricetes; Durnavirales*	Picobirnaviridae	*Picobirnavirus*	human picobirnavirus
(+)RNA			*Pisoniviricetes; Nidovirales*	*Coronaviridae*; *Orthocoronavirinae*	*Betacoronavirus*	SARS-CoV-2 ^2^
			*Pisoniviricetes; Picornavirales*	*Picornaviridae*	*Kobuvirus*	aichivirus C
					*Cardiovirus*	cardiovirus A
					*Aphthovirus*	foot-and-mouth disease virus
					*Enterovirus*	enterovirus A71 (enterovirus A)
						coxsackievirus B3 (enterovirus B)
						poliovirus 1 (enterovirus C)
						human rhinovirus A
					*Sicinivirus*	sicinivirus A
				*Caliciviridae*	*Norovirus*	Norwalk virus
					*Lagovirus*	rabbit hemorrhagic disease virus
					*Sapovirus*	Sapporo virus
		*Lenarviricota*	*Allassoviricetes*; *Levivirales*	*Leviviridae*	*Allolevivirus*	Enterobacteria phage Qβ

^1^ According to ICTV Virus Taxonomy: 2019 release (https://talk.ictvonline.org/taxonomy/, 11 November 2020). ^2^ Severe acute respiratory syndrome coronavirus 2; *Coronaviridae* Study Group of the International Committee on Taxonomy of Viruses [42].

## Data Availability

Not applicable.

## Supplementary Materials

The following are available online at https://www.mdpi.com/1999-4915/13/2/313/s1, Figure S1: RdRp subdomains, sequence motifs, homomorphs and structurally equivalent residues mapped on the amino acid sequence of poliovirus RdRp, Figure S2: Coverage of sequence motifs A–G by the structurally equivalent residues in viral RNA-dependent RNA polymerases (RdRps), Figure S3: Consensus and jackknifing trees, Table S1: The Protein Data Bank identifiers, chains, resolutions, lengths in amino acids, and references for protein structures used in the study, Table S2: HSF parameters and applied values, Table S3: The Ete-compare tool comparison of jackknifing trees, Supplementary Data: Amino acid sequence alignment for RdRps used in the study.
