# Supplementary material for: Structure Unveils Relationships between RNA Virus Polymerases
Source: Viruses. 2021 Feb 17;13(2):313. doi: 10.3390/v13020313 (PMC7922027; doi:10.3390/v13020313)
Supplement: Supplementary file 1 [file viruses-13-00313-s001.zip › Supplementary File.pdf]

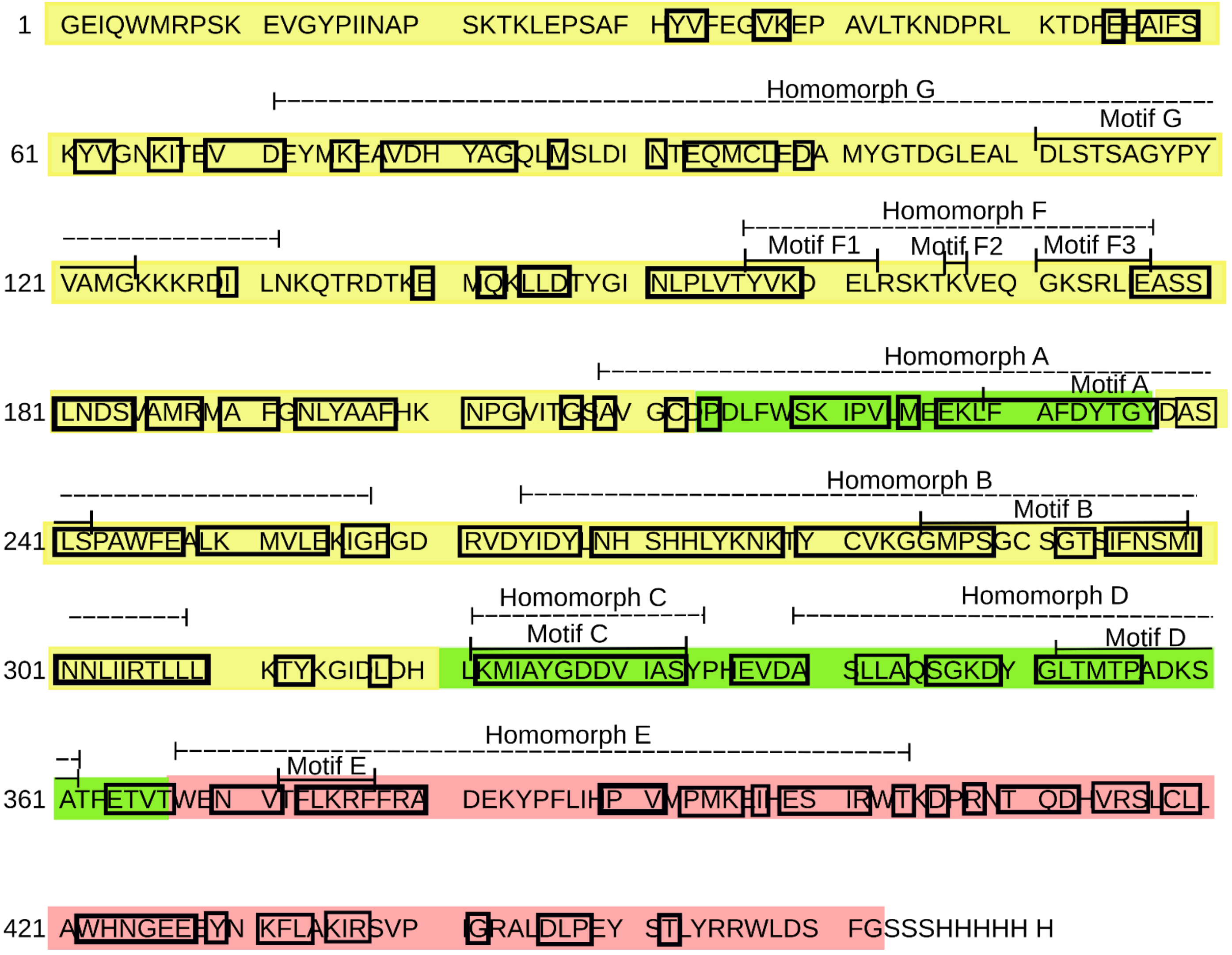

Supplementary Figure S1. RdRp subdomains, sequence motifs, homomorphs and structurally equivalent residues mapped on the amino acid sequence of poliovirus RdRp. The structurally equivalent residues comprising the identified RdRp core structure are marked with black squares. The fingers, palm and thumb subdomains of RdRps are marked with yellow, palm and thumb, respectively. The sequence motifs (solid line) and homomorphs (dashed line) are according to Lang et al., 2013 [20]. Supplementary

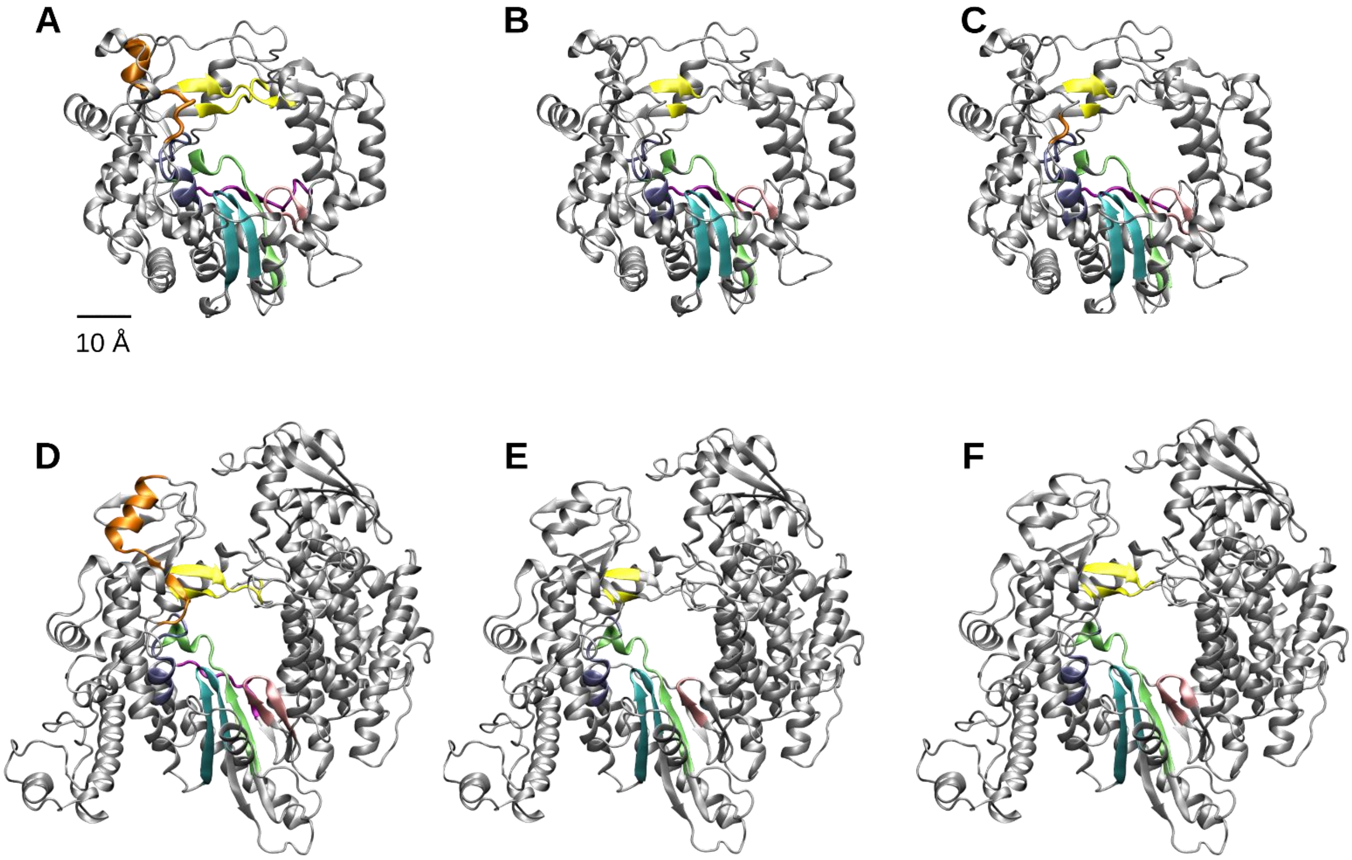

Supplementary Figure S2. Coverage of sequence motifs A–G by the structurally equivalent residues in viral RNA-dependent RNA polymerases (RdRps). The motifs are indicated with different colours on the poliovirus (A–C) and vesicular stomatitis Indian virus (D–F) RdRp structures [Protein Data Bank identifier: 3OL6 and 5A22 (residues 35–865 of the chain A), respectively]. (A and C) The full-length motifs A–G. (B and E) The structurally equivalent residues identified for all RdRps in the dataset that overlap with the sequence motifs. (C and F) The structurally equivalent residues identified for Cluster II and Cluster I RdRps, respectively, which overlap with the motifs. The motifs are indicated as motif A: lime, motif B: ice blue, motif C: cyan, motif D: purple, motif E: pink, motif F: yellow and motif G: orange.

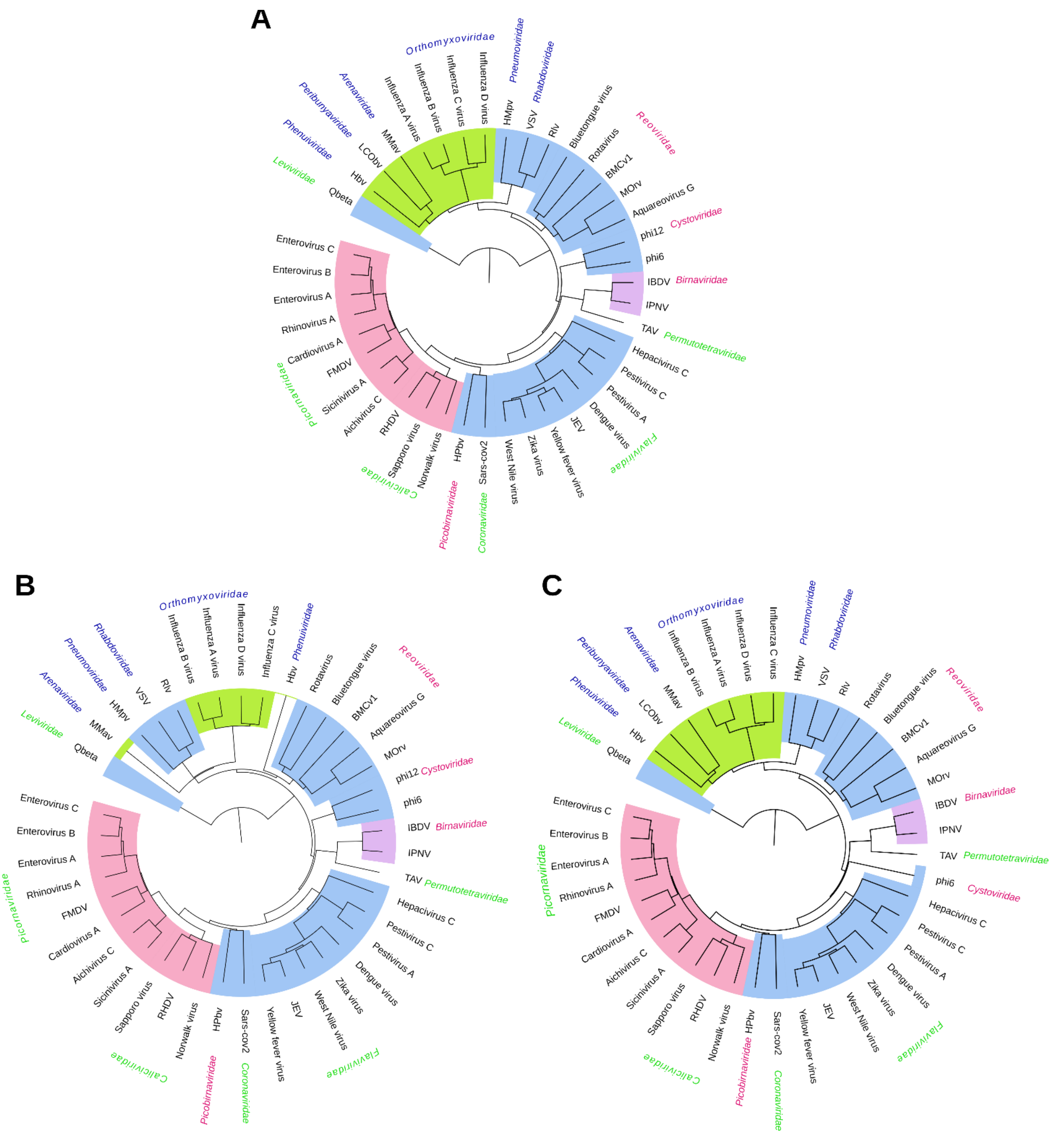

Supplementary Figure S3. Consensus and jackknifing trees. A consensus tree (A) that is calculated from all jackknifing trees and jackknifing trees from which either (B) a RdRp from a member of *Peribunyaviridae* family or (C) a member of *Cystoviridae* family are removed. (B) and (C) are the only jackknifing trees which differed from the original tree calculated for the whole dataset (shown in Figure 2) or the jackknifing consensus tree in panel (A). The applied abbreviations are: BMCv2: Bombyx Mori cypovirus 1, FMDV: foot-and-mouth disease virus, Hbv: Huaiyangshan banyangvirus, HPbv: human picobirnavirus, HMpv: human metapneumovirus, IBDV: infectious bursal disease virus, IPNV: infectious pancreatic necrosis virus, JEV: Japanese encephalitis virus, LCObv: La Crosse orthobunyavirus, MMav: Machupo mammarenavirus, MOrv: mammalian orthoreovirus, Qbeta: Escherichia phage Qbeta, RHDV: rabbit haemorrhagic disease virus, Rlv: rabies lyssavirus, SARS-cov2: severe acute respiratory syndrome coronavirus 2, TAV: thosean asigna virus, VSV: vesicular stomatitis Indian virus. The coloring of the family name reflects the nature of the viral genome: green, (+)RNA; blue, (-)RNA; and red, double-stranded RNA. The polymerases using solely primer-independent initiation are indicated with blue background, polymerases dependent on VPg-protein primer with pink background, polymerases applying de novo initiation on (-)strand and self-priming on (+)strand are shown with purple background and polymerases using de novo initiation on (-)strand and RNA-priming on (+)strand synthesis with green background. Background is white if the initiation mechanism is unclear.

Supplementary table S1. The Protein Data Bank (PDB) identifiers, chains, resolutions, lengths in amino acids, and references for protein structures used in the study.

Genome type	Family	Species	PDBid	Chain	Resolution (Å)	Length in amino acids (according to PDB)	Amino acids in the PDB structure	Reference
Negative-strand RNA	*Arenaviridae*	Machupo mammarenavirus	6KLD*	A	3.58	2209	1587	Peng R, Xu X, Jing J, Wang M, Peng Q, Liu S, Wu Y, Bao X, Wang P, Qi J, Gao GF, Shi Y. Structural insight into arenavirus replication machinary. *Nature* **2020**, *579*, 615-619.
	Orthomyxoviridae	influenza A virus	4WSB	B	2.65	776	745	Reich S, Guilligay D, Pflug A, Malet H, Berger I, Crepin T, Hart D, Lunardi T, Nanao M, Ruigrok RW, et al. Structural insight into cap-snatching and RNA synthesis by influenza polymerase. *Nature* **2014**, *516*, 361-366.
		influenza B virus	4WRT	B	2.7	772	742	Reich S, Guilligay D, Pflug A, Malet H, Berger I, Crepin T, Hart D, Lunardi T, Nanao M, Ruigrok RW, et al. Structural insight into cap-snatching and RNA synthesis by influenza polymerase. *Nature* **2014**, *516*, 361-366.
		influenza C virus	5D98*	A	3.9	754	711	Hengrung N, El Omari K, Serna Martin I, Vreede FT, Cusack S, Rambo RP, Vonrhein C, Bricogne G, Stuart DI, Grimes JM, et al. Crystal structure of the RNA-depedent RNA polymerase from influenza C virus. *Nature* **2015** *527*, 114-117.
		influenza D virus	6KUJ*	A	3.4	753	705	Peng Q, Liu Y, Peng R, Wang M, Yang W, Song H, Chen Y, Liu S, Han M, Zhang X, et al. Structure of influenza D virus polymerase bound to cRNA promoter in mode A conformation. *Nat. Microbiol.* **2019**, *4*, 1750-1759.
	Peribunyaviridae	La Crosse orthobunyavirus	5AMQ	A	3	2263	1666	Gerlach P, Malet H, Cusack S, Reguera J. Structural insight into bunyavirus replication and its regulation by Vrna Promoter. *Cell* **2015** *161*, 1267-1279.
	Phenuiviridae	Huaiyangshan banyangvirus	6Y6K*	A	3.78	2084	1300	Vogel D, Thorkelsson SR, Quemin ERJ, Meier K, Kouba T, Gogrefe N, Busch C, Reindl S, Gunther S, Cusack S, et al. Structural and functional characterization of the severe fever with thrombocytopenia syndrome virus L protein. *Nucleic Acids Res*. **2020**, *48,* 5749-5765.
	Pneumoviridae	human metapneumovirus	6U5O*	L	3.7	2030	1354	Pan, J., Qian, X., Lattmann, S., El Sahili, A., Yeo, T.H., Jia, H., Cressey, T., Ludeke, B., Noton, S., Kalocsay, M., Fearns, R., Lescar, J. Structure of the human metapneumovirus polymerase phosphoprotein complex. *Nature* **2020**, 577, 275-279.
	Rhabdoviridae	rabies lyssavirus	6UEB*	A	3.3	2127	2099	Horwitz JA, Jenni S, Harrison SC, Whelan SPJ. Structure of a rabies virus polymerase complex from electro cryo-microscopy. *Proc. Natl. Acad. Sci USA* **2020**, 117, 2099-2107.
		vesicular stomatitis Indian virus	5A22*	A	3.8	2109	2002	Liang B, Li Z, Jenni S, Rahmeh AA, Morin BM, Grant T, Grigorieff N, Harrison SC, Whelan SP. Structure of the L protein of vesicular stomatitis virus from electron cryomicroscopy. *Cell* **2015**, *162*, 314.
Positive-strand RNA	*Caliciviridae*	Norwalk virus	3BSO	A	1.74	510	482	Zamyatkin DF, Parra F, Alonso JM, Harki DA, Peterson BR, Grochulski P, Ng KK. Structural insights into mechanisms of catalysis and inhibition in norwalk virus polymerases. *J. Biol. Chem.* **2008**, *283*, 7705-7712.
		rabbit haemorrhagic disease virus	1KHV	A	2.5	516	493	Ng KK, Cherney MM, Vazquez AL, Machin A, Alonso JM, Parra F, James MN. Crystal structures of active and inactive conformations of a caliciviral RNA-dependent RNA polymerase. *J. Biol: Chem.* **2002**, *277*, 1381-1387.
		Sapporo virus	2UUT	A	2.4	508	492	Fullerton SWB, Robel I, Schuldt L, Gebhardt J, Tucker P, Rohayem J.
	Coronaviridae	Severe acute respiratory syndrome coronavirus 2	7BTF*	A	2.95	942	924	Gao Y, Yan L, Huang Y, Liu F, Zhao Y, Cao L, Wang T, Sun Q, Ming Z, Zhang L, et al. Structure of the RNA-dependent RNA polymerase from COVID-19 virus. *Science* **2020**, *368*, 779-782.
	Flaviviridae	Dengue virus	3VWS	A	2.1	635	592	Noble CG, Lim SP, Chen, YL, Liew, CW, Yap L, Lescar J, Shi P-Y. Conformational flexibility of the Dengue virus RNA-dependent RNA polymerase revealed by a complex with an inhibitor. *J Virol.* **2013**, *87*, 5291-5295.
		hepacivirus C	3TYQ	A	1.6	576	563	Chen KX, Lesburg CA, Vibulbhan B, Yang W, Chan TY, Venkatraman S, Velazquez F, Zeng Q, Bennett F, Anilkumar GN. et al. A Novel Class of Highly Potent Irreversible Hepatitis C Virus NS5B Polymerase Inhibitors. *J. Med. Chem.* **2012**, *55*, 2089-2101.
		Japanese encephalitis virus	4K6M	A	2.6	915	888	Lu, G., Gong, P. Crystal Structure of the full-length Japanese encephalitis virus NS5 reveals a conserved methyltransferase-polymerase interface. *PLoS Pathog*. **2013**, *9*, e1003549-e1003549.
		pestivirus A	1S49	A	3	609	576	Choi, K.H., Groarke, J.M., Young, D.C., Kuhn, R.J., Smith, J.L., Pevear, D.C., Rossmann, M.G. The structure of the RNA-dependent RNA polymerase from bovine viral diarrhea virus establishes the role of GTP in de novo initiation. *Proc. Natl. Acad. Sci. USA* **2004**, *101*, 4425-4430.
		pestivirus C	6AE5*	A	2.75	683	644	Liu W, Shi X, Gong P. A unique intra-molecular fidelity-modulating mechanism identified in a viral RNA-dependent RNA polymerase. *Nucleic Acids Res*. **2018**, *46,* 10840-10854.
		West Nile virus	2HFZ	A	3	639	608	Malet H, Egloff MP, Selisko B, Butcher RE, Wright PJ, Roberts M, Gruez A, Sulzenbacher G, Vonrhein C, Bricogne G, et al. Crystal structure of the RNA polymerase domain of the West Nile virus non-structural protein 5. *J. Biol. Chem.* **2007**, *282*, 10678-10689.
		Yellow fever virus	6QSN*	A	3	905	882	Dubankova A, Boura E. Structure of the yellow fever NS5 protein reveals conserved drug targets shared among flaviviruses. *Antiviral. Res.* **2019**, *169*, 104536-104536
		Zika virus	5WZ3	A	1.8	619	565	Duan W, Song H, Wang H, Chai Y, Su C, Qi J, Shi Y, Gao GF. The crystal structure of Zika virus NS5 reveals conserved drug targets. *EMBO J*. **2017,** *36*, 919-933.
	Leviviridae	phage Qβ	3MMP	F	2.5	589	543	Kidmose RT, Vasiliev NN, Chetverin AB, Andersen GR, Knudsen CR. Structure of the Qbeta replicase, an RNA-dependent RNA polymerase consisting of viral and host proteins. *Proc. Natl. Acad. Sci. USA* **2010**, *107*, 10884-10889.
	Permutotetraviridae	thosean asigna virus	5CYR	A	3.5	705	662	Ferrero DS, Buxaderas M, Rodriguez JF, Verdaguer N. The structure of the RNA-dependent RNA polymerase of a permutotetravirus suggests a link between primer-dependent and primer-independent polymerases. *PloS Pathog.* **2015**, *11*, e1005265-e1005265.
	Picornaviridae	aichivirus	6R1I*	A	2.63	468	468	Dubankova A, Horova V, Klima M, Boura E. Structures of kobuviral and siciniviral polymerases reveal conserved mechanism of picornaviral polymerase activation. *J. Struct. Biol.* **2019**, *208*, 92-98.
		coxsackievirus B3 (enterovirus B)	3CDU	A	2.1	468	468	Gruez A, Selisko B, Roberts M, Bricogne G, Bussetta C, Jabafi I, Coutard B, De Palma AM, Neyts J, Canard B. The crystal structure of coxsackievirus B3 RNA-dependent RNA polymerase in complex with its protein primer VPg confirms the existence of a second VPg binding site on *Picornaviridae* polymerases. *J. Virol*. **2008**, *82*, 9577-9590.
		cardiovirus A	4NYZ	A	2.15	460	460	Vives-Adrian L, Lujan C, Oliva B, van der Linden L, Selisko B, Coutard B, Canard B, van Kuppeveld FJ, Ferrer-Orta C, Verdaguer N. The crystal structure of a cardiovirus RNA-dependent RNA polymerase reveals an unusual conformation of the polymerase active site. *J. Virol*. **2014**, *88*, 5595-5607.
		foot-and-mouth disease virus	1U09	A	1.91	476	476	Ferrer-Orta C, Arias A, Perez-Luque R, Escarmis C, Domingo E, Verdaguer N. Structure of Foot-and-Mouth Disease Virus RNA-dependent RNA Polymerase and Its Complex with a Template-Primer RNA. *J. Biol. Chem*. **2004**, *279*, 47212-47221
		human enterovirus A71 (enterovirus A)	3N6M	A	2.5	462	462	Wu Y, Lou Z, Miao Y, Yu Y, Dong H, Peng W, Bartlam M, Li X, Rao Z. Structures of EV71 RNA-dependent RNA polymerase in complex with substrate and analogue provide a drug target against the hand-foot-and-mouth disease pandemic in China. *Protein Cell* **2010**, *1*, 491-500.
		Poliovirus 1 (enterovirus C)	3OL6	A	2.5	471	461	Gong P, Peersen OB. Structural basis for active site closure by the poliovirus RNA-dependent RNA polymerase. *Proc. Natl. Acad. Sci. USA* **2010**, *107*, 22505-22510.

		human rhinovirus A	1TP7	A	2.4	460	450	Appleby TC, Luecke H, Shim JH, Wu JZ, Cheney IW, Zhong W, Vogeley L, Hong Z, Yao N. Crystal structure of complete rhinovirus RNA polymerase suggests front loading of protein primer. *J. Virol.* **2005**, *79*, 277-288.
		sicinivirus A	6QWT*	A	2.3	472	471	Dubankova, A., Horova, V., Klima, M., Boura, E. Structures of kobuviral and siciniviral polymerases reveal conserved mechanism of picornaviral polymerase activation. *J. Struct. Biol*. **2019**, *208*, 92-98.
Double-stranded RNA	*Birnaviridae*	infectious bursal disease virus	2YI9	A	2.2	799	771	Garriga D, Navarro A, Querol-Audi J, Abaitua F, Rodriguez JF, Verdaguer N. Activation mechanism of a noncanonical RNA-dependent RNA polymerase. *Proc. Natl. Acad. Sci. USA* **2007**, *104*, 20540-20545.
		infectious pancreatic disease virus	2PUS	A	2.4	852	765	Graham SC, Sarin LP, Bahar MW, Myers RA, Stuart DI, Bamford DH, Grimes JM. The N-Terminus of the RNA Polymerase from Infectious Pancreatic Necrosis Virus is the Determinant of Genome Attachment. *PLoS Pathog.* **2011**, *7*, 2085.
	Cystoviridae	Pseudomonas phage phi12	4GZK	A	1.69	659	650	Ren Z, C Franklin M, Ghose R. Structure of the RNA-directed RNA Polymerase from the cystovirus 12. *Proteins* **2013**, *81*, 1479-1484.
		Pseudomonas phage phi6	1UVJ	A	1.9	664	664	Salgado PS, Makeyev EV, Butcher SJ, Bamford DH, Stuart DI, Grimes JM. The structural basis for RNA specificity and Ca2+ inhibition of an RNA-dependent RNA polymerase. *Structure* **2002**, *12*, 307-316.
	Picobirnaviridae	human picobirnavirus	5I61	A	2.4	534	505	Collier AM, Lyytinen OL, Guo YR, Toh Y, Poranen MM, Tao YJ. Initiation of RNA Polymerization and Polymerase Encapsidation by a Small dsRNA Virus. *PLoS Pathog*. **2016**, *12*, e1005523-e1005523.
	Reoviridae	aquareovirus G	5ZVS*	A	3.8	1214	1060	Wang X, Zhang F, Su R, Li X, Chen W, Chen Q, Yang T, Wang J, Liu H, Fang Q, et al. Structure of RNA polymerase complex and genome within a dsRNA virus provides insights into the mechanisms of transcription and assembly. *Proc. Natl. Acad. Sci. USA* **2018**, *115*, 7344-7349.
		bluetongue virus	6PNS*	A	3.7	1302	1291	He Y, Shivakoti S, Ding K, Cui Y, Roy P, Zhou ZH. In situ structures of RNA-dependent RNA polymerase inside bluetongue virus before and after uncoating. *Proc. Natl. Acad. Sci. USA* **2019**, *116*, 16535-16540.
		Bombyx mori cypovirus 1	3JB6*	A	3.3	1225	1196	Zhang X, Ding K, Yu X, Chang W, Sun J, Hong Zhou Z. In situ structures of the segmented genome and RNA polymerase complex inside a dsRNA virus. *Nature* **2015**, *527*, 531-534.
		mammalian orthoreovirus	1MWH	A	2.5	1267	1256	Tao Y, Farsetta DL, Nibert ML, Harrison SC. RNA Synthesis in a Cage-Structural Studies of Reovirus Polymerase lambda3. *Cell* **2002**, 111, 733-745.
		rotavirus A	2R7R	A	2.6	1095	1073	Lu X, McDonald SM, Tortorici MA, Tao YJ, Vasquez-Del Carpio R, Nibert ML, Patton JT, Harrison SC. Mechanism for coordinated RNA packaging and genome replication by rotavirus polymerase VP1. *Structure* **2008**, *16,* 1678-1688.

*Protein structures from virus species not present in Venkataraman, S.; Prasad, B.; Selvarajan, R. RNA dependent RNA polymerases: Insights from structure, function and evolution. Viruses **2018**, 10, 76, doi:10.3390/v10020076 or Jácome, R.; Becerra, A.; Ponce de León, S.; Lazcano, A. Structural analysis of monomeric RNA-dependent polymerases: Evolutionary and therapeutic implications. PLoS One **2015**, 10, e0139001, doi:10.1371/journal.pone.0139001.

Supplementary Table S2. HSF parameters and applied values

Property		Weight (Scaled to 100)
Geometry		
	Half sphere exposure (HSE) Cα-Cβ	3.633
	HSE down	3.386
	HSE up	2.979
	Dihedral angular φ	3.211
	Dihedral angular ψ	2.950
	Distance to center of gravity	1.133
	Density	3.488
	Moment of inertia	1.105
	Local geometry[1]	22.000
Secondary structure		
	Secondary structure type	2.572
	Position in secondary structure	2.921
Sequence		
	Amino acid type	1.409
	Position in sequence	3.430
Physicochemical properties of amino acids		
	Hydroxylic	1.206
	Hydrophobic	2.471
	Aliphatic	1.395
	Aromatic	1.148
	Charged	1.410
	Negative	1.192
	Positive	1.177
	Polar	1.991
	Small	1.890
	Tiny	1.279
	Turn-like	2.485
Variable		
	Cα-Cα distance	5.610
	Backbone direction	1.293
Other		
	Local alignment[2]	21.247

[1]Local geometry is defined on a ±4 residue-window around a given residue. The similarity is defined as a sum of differences of distance matrices calculated from Cα-coordinates of each window within each pair of residues from each compared core.

[2]Dynamic programming module is used to further detect local agreements of the similarities (e.g. improving alignment of helices between cores). The module produces a new reweighted residue similarity matrix from the equivalences according to given parameters (match, mismatch and gap).

Supplementary Table S3. The Ete-compare tool comparison of jackknifing trees

				Ete-compare output					
Virus family with omitted member	Omitted structure	Size of core	RMSD (Ångströms)	Effective tree size	Normalized Robinson-Foulds distance	Robinson-Foulds symmetric distance	Maximum Robinson-Foulds value for this distance	Frequency of edges in target tree found in the reference (%)	Frequency of edges in the reference tree found in target (%)
Arenaviride	6KLD	240	4.49	41	0.00	0.00	78.00	1.00	1.00
Birnaviridae	2YI9	228	4.55	41	0.00	0.00	78.00	1.00	1.00
Caliciviridae	1KHV	231	4.56	41	0.00	0.00	78.00	1.00	1.00
Coronaviridae	7BTF	228	4.53	41	0.00	0.00	78.00	1.00	1.00
Cystoviridae	4GZK	227	4.43	41	0.03	2.00	78.00	0.97	0.97
Flaviviridae	3VWS	234	4.54	41	0.00	0.00	78.00	1.00	1.00
Leviviridae	3MMP	230	4.45	41	0.00	0.00	78.00	1.00	1.00
Orthoviridae	5D98	230	4.5	41	0.00	0.00	78.00	1.00	1.00
Peribunyaviridae	5AMQ	228	4.64	41	0.10	8.00	78.00	0.90	0.90
Permutotetraviridae	5CYR	224	4.54	41	0.00	0.00	78.00	1.00	1.00
Phenuiviridae	6Y6K	219	4.8	41	0.00	0.00	78.00	1.00	1.00
Picobirnaviridae	5I61	227	4.5	41	0.00	0.00	78.00	1.00	1.00
Picornaviridae	4NYZ	234	4.56	41	0.00	0.00	78.00	1.00	1.00
Pneumoviridae	6U5O	230	4.53	41	0.00	0.00	78.00	1.00	1.00
Reoviridae	3JB6	232	4.49	41	0.00	0.00	78.00	1.00	1.00
Rhabdoviridae	5A22	228	4.46	41	0.00	0.00	78.00	1.00	1.00
